# Development and validation of prognostic nomograms for patients with colon neuroendocrine neoplasms

**DOI:** 10.1186/s12957-021-02338-8

**Published:** 2021-08-07

**Authors:** Ruitong Xu, Bingrong Zhou, Ping Hu, Bingyan Xue, Danyang Gu, Xiaolin Li, Qiyun Tang

**Affiliations:** 1 Department of Geriatric Gastroenterology, the First Affiliated Hospital of Nanjing Medical University, Gulou District, No. 300, Guangzhou Road, Nanjing, 210029 China; 2 Department of Dermatology, the First Affiliated Hospital of Nanjing Medical University, Gulou District, No. 300, Guangzhou Road, Nanjing, 210029 China

**Keywords:** Colon neuroendocrine neoplasm, Nomogram, Prognosis, Survival

## Abstract

**Background:**

Colon neuroendocrine neoplasms (NENs) have one of the poorest median overall survival (OS) rates among all NENs. The American Joint Committee on Cancer (AJCC) tumor–node–metastasis (TNM) staging system—currently the most commonly used prediction model—has limited prediction accuracy because it does not include parameters such as age, sex, and treatment. The aim of this study was to construct nomograms containing various clinically important parameters to predict the prognosis of patients with colon NENs more accurately.

**Methods:**

Using the Surveillance, Epidemiology, and End Results (SEER) database, we performed a retrospective analysis of colon NENs diagnosed from 1975 to 2016. Data were collected from 1196 patients; almost half were female (617/1196, 51.6%), and the average age was 61.94 ± 13.05 years. Based on the age triple cut-off values, there were 396 (33.1%), 408 (34.1%), and 392 (32.8%) patients in age groups 0–55 years, 55–67 years, and ≥ 68 years, respectively. Patients were randomized into training and validation cohorts (3:1). Independent prognostic factors were used for construction of nomograms to precisely predict OS and cancer-specific survival (CSS) in patients with colon NENs.

**Results:**

Multivariate analysis showed that age ≥ 68 years, sex, tumor size, grade, chemotherapy, N stage, and M stage were independent predictors of OS. In the validation cohort, the Concordance index (C-index) values of the OS and CSS nomograms were 0.8345 (95% confidence interval [CI], 0.8044–0.8646) and 0.8209 (95% CI, 0.7808–0.861), respectively. C-index also indicated superior performance of both nomograms (C-index 0.8347 for OS and 0.8668 for CSS) compared with the AJCC TNM classification (C-index 0.7159 for OS and 0.7366 for CSS).

**Conclusions:**

We established and validated new nomograms for more precise prediction of OS and CSS in patients with colon NENs to facilitate individualized clinical decisions.

**Supplementary Information:**

The online version contains supplementary material available at 10.1186/s12957-021-02338-8.

## Background

Neuroendocrine neoplasms (NENs) are a heterogeneous group of rare tumors derived from peptidergic neurons and neuroendocrine cells of the diffuse neuroendocrine system. According to tumor differentiation, NENs include well or moderately differentiated neuroendocrine tumor (NET), poorly differentiated neuroendocrine carcinoma (NEC), and mixed neuroendocrine non-neuroendocrine neoplasm (MiNEN). Biological behavior of NET is relatively benign compared with NEC or mixed adeno-neuroendocrine carcinoma (MANEC) [1]. A population-based study from nationally representative data from the Surveillance, Epidemiology, and End Results (SEER) program showed that the incidence of NENs has increased from 1.09/100,000 in 1973 to 6.98/100,000 in 2012 [2]. The gastroenteropancreatic tract is the most common site of extrapulmonary NENs. Among them, NENs originating from the cecum to the sigmoid colon account for 4–8% of all NENs [2–6]. Although colon and rectal NENs are often described as a single disease, recent evidence has indicated that depending on tumor grade, the median overall survival (OS) of colon NENs is poor compared with most gastrointestinal NENs, and the prognosis is much worse than that of rectal NENs [2, 3]. Compared with rectal NENs, colon NENs have larger size, higher T stage, higher grade, and more frequent lymph nodes and lymphovascular invasion positivity. The 5-year disease-specific survival is also significantly different, and treatment options are very limited, which leads to worse outcomes [1, 7, 8].

The American Joint Committee on Cancer (AJCC) and the World Health Organization (WHO) have proposed systems to predict the prognosis of colon NENs [9, 10]. The AJCC tumor–node–metastasis (TNM) staging system includes T stage, N stage, and M stage, whereas the WHO classification includes the mitotic count and Ki-67 proliferation index. Currently, these systems are the most commonly used prediction models for colon NENs, but they contain only two to three parameters and do not incorporate certain relevant parameters, such as age, sex, and treatment [11], which are equally important for the prognosis of patients with colon NENs. Therefore, there is an urgent need for a usable decision tool that can integrate additional parameters to assist with clinical practice, decision-making, and accurate prediction in patients with colon NENs.

Nomograms-graphical calculations or algorithms with continuous scales to calculate the probability of a particular outcome-have recently been shown to be a more effective method for predicting the prognosis of various cancers than traditional staging systems [12–15]. However, no studies have established a prognostic nomogram to predict the outcomes of patients with colon NENs.

To the best of our knowledge, this study is the first attempt to develop nomograms for colon NENs based on a retrospective study of the SEER database that incorporates additional clinical parameters to predict survival more accurately.

## Materials and methods

### Data retrieved from the SEER database

Specific clinicopathological data and prognostic outcomes of patients with colon NENs were retrieved from the SEER database submitted in November 2018 [16]. This study did not require a local ethics approval or statement because all the data were publicly available. The International Classification of Diseases for Oncology (ICD-O-3) was used to identify cases of colon NENs. The primary site codes (C18.0, C-18.2-C18.9, colon) and the following ICD-O-3 codes for histological type were used to identify cases with colon NENs: large cell neuroendocrine carcinoma (8013), small cell carcinoma (8041), carcinoid tumor (8240), enterochromaffin cell carcinoid (8241), neuroendocrine carcinoma (8244), mixed adeno-neuroendocrine carcinoma (MANEC) (8246), and atypical carcinoid tumor (8249). The inclusion criteria were as follows: (I) complete TNM stage information available; (II) only one primary tumor lesion; (III) all data classified using the new 8th Edition the AJCC staging system; (IV) complete survival data available; (V) no missing data in the SEER other cause of death classification; (VI) known tumor size; and (VII) known grade. The exclusion criteria were as follows: (I) the presence of other tumors at the same time; (II) unknown stage; and (III) incomplete clinical data (e.g., tumor size, treatment, grade). These eligible patients with colon NENs were subsequently randomly assigned into a training cohort and a validation cohort. In addition, the patients were grouped based on age into three categories (0–55 years, 56–67 years, and ≥ 68 years); they were also grouped based on the size of the tumor into < 35 mm and ≥ 35 mm groups.

### Study variables

We retrieved the following demographic or clinical variables from the SEER database: age, sex, tumor grade, tumor site, tumor size, AJCC TNM stage, surgery and chemotherapy, SEER other cause of death classification, and survival-related information. The stage classification was in accordance with the criteria of the 8th AJCC TNM staging system. The primary end point was OS, whereas the secondary end point was cancer-specific survival (CSS).

### Construction and validation of nomogram model

The entire sample was randomized into two groups: 896 (approximately 75%) cases were included in the training cohort, and 300 (approximately 25%) in the validation cohort. At diagnosis, patient age (years) and survival time (months) are expressed as mean ± SD or median (25th–75th percentile) depending on the data distribution. The Mann–Whitney *U* test was used for the analysis of continuous variables, and the chi-square test was used for comparison of categorical variables between groups of patients who survived or those who did not. Univariate and multivariate Cox proportional-risk regression analyses were used to assess factors associated with OS and CSS. By using univariate Cox analysis to determine the potential prognostic factors associated with OS and CSS of colon NENs, variables with *P* < 0.05 were included in the final multivariate analysis and construction of the nomogram. The receiver operating characteristic (ROC) curves were analyzed by measuring the performance of the constructed nomogram. The accuracy of the nomogram was analyzed using the Harrell C-index and the area under the time-dependent ROC curve (AUC). A larger C-index is associated with better predictive ability of the nomogram [17]. Calibration curves were plotted to assess the agreement between the nomogram-predicted survival rate and the observed survival rate. Furthermore, C-index of the nomogram was compared with C-index of the TNM stage. In summary, the nomograms were constructed to predict the survival of colon NENs patients. All analyses were performed using the statistical package R (http://www.r-project.org) and Empower Stats software (http://www.empowerstats.com, X&Y Solutions, Inc., Boston, MA). Statistical significance was set at 0.05.

## Results

### Baseline characteristics of the patients

A total of 1196 patients with colon NENs from the SEER database were included in the study, with 896 patients in the training cohort and 300 patients in the validation cohort. The proportions of female and male patients were 51.6% and 48.4%, respectively. Grades G1, G2, G3, and G4 accounted for 39.8%, 16%, 29.1%, and 15.1% of cases, respectively. The primary site was in the right hemicolon in 76.5% of cases, while in 23.5% of cases the tumor was located in the left hemicolon. The median tumor size was 41.0 mm. Other characteristics are presented in Table 1. Overall, patients in the training and validation cohorts were comparable in terms of demographic and clinicopathological features (Table 1).

**Table 1:** Demographic and clinical characteristics of patients in the training and validation cohorts

Variable	Training cohort (*n* = 896)	Validation cohort (*n* = 300)	Total (*n* = 1196)	*P* value
Median age, years	61.8 ± 13.0	62.3 ± 13.1	61.9 ± 13.1	0.599
Age, years				0.666
0–55	33.8% (303/896)	31% (93/300)	33.1% (396/1196)	
56–67	33.8% (303/896)	35% (105/300)	34.1% (408/1196)	
≥ 68	32.4% (290/896)	34% (102/300)	32.8% (392/1196)	
Sex				0.919
Female	51.7% (463/896)	51.3% (154/300)	51.6% (617/1196)	
Male	48.3% (433/896)	48.7% (146/300)	48.4% (579/1196)	
Median tumor diameter, mm	40.5 ± 28.1	42.6 ± 38.7	41 ± 31.1	0.3
Tumor size, mm				0.843
< 35	48.3% (433/896)	47.7% (143/300)	48.2% (576/1196)	
≥ 35	51.7% (463/896)	52.3% (157/300)	51.8% (620/1196)	
Laterality				0.812
Right	76.7% (687/896)	76% (228/300)	76.5% (915/1196)	
Left	23.3% (209/896)	24% (72/300)	23.5% (281/1196)	
Grade				0.031
G1	39.8% (357/896)	39.7% (119/300)	39.8% (476/1196)	
G2	15.5% (139/896)	17.7% (53/300)	16.1% (192/1196)	
G3	30.9% (277/896)	23.7% (71/300)	29.1% (348/1196)	
G4	13.7% (123/896)	19% (57/300)	15.1% (180/1196)	
Pathology				0.006
8013/3 large cell neuroendocrine carcinoma	7.5% (67/896)	7.7% (23/300)	7.5% (90/1196)	
8041/3 small cell carcinoma	4.1% (37/896)	3.3% (10/300)	3.9% (47/1196)	
8240/3 carcinoid tumor	37.9% (340/896)	34.7% (104/300)	37.1% (444/1196)	
8244/3 mixed adeno-neuroendocrine carcinoma	3.8% (34/896)	9.3% (28/300)	5.2% (62/1196)	
8246/3 neuroendocrine carcinoma	46.7% (418/896)	45% (135/300)	46.2% (553/1196)	
Operation				0.864
No operation	3.7% (33/896)	3.3% (10/300)	3.6% (43/1196)	
Local tumor excision	8.5% (76/896)	7.7% (23/300)	8.3% (99/1196)	
Curative	87.8% (787/896)	89% (267/300)	88.1% (/1196)	
Chemotherapy				0.82
No	72% (645/896)	72.7% (218/300)	72.2% (863/1196)	
Yes	28% (251/896)	27.3% (82/300)	27.8% (333/1196)	
AJCC TNM stage (8th)				0.337
I	12.2% (109/896)	10.3% (31/300)	11.7% (140/1196)	
IIA	2.6% (23/896)	1.7% (5/300)	2.3% (28/1196)	
IIB	6.5% (58/896)	9.3% (28/300)	7.2% (86/1196)	
IIIA	1.2% (11/896)	2% (6/300)	1.4% (17/1196)	
IIIB	42% (376/896)	44% (132/300)	42.5% (508/1196)	
IV	35.6% (319/896)	32.7% (98/300)	34.9% (417/1196)	
AJCC T stage (8th)				0.066
T1	0.6% (5/896)	0.7% (2/300)	0.6% (7/1196)	
T1a	10.7% (96/896)	9.3% (28/300)	10.4% (124/1196)	
T1b	3.7% (33/896)	2.3% (7/300)	3.3% (40/1196)	
T2	11.6% (104/896)	8% (24/300)	10.7% (128/1196)	
T3	49.9% (447/896)	48% (144/300)	49.4% (591/1196)	
T4	23.5% (211/896)	31.7% (95/300)	25.6% (306/1196)	
AJCC N stage (8^th^)				0.968
N0	26.8% (240/896)	26.7% (80/300)	26.8% (320/1196)	
N1	73.2% (656/896)	73.3% (220/300)	73.2% (876/1196)	
AJCC M stage (8^th^)				0.356
M0	64.4% (577/896)	67.3% (202/300)	65.1% (779/1196)	
M1	35.6% (319/896)	32.7% (98/300)	34.9% (417/1196)	
OS				0.897
Alive	53.2% (477/896)	53.7% (161/300)	53.3% (638/1196)	
Dead	46.8%(419/896)	46.3% (139/300)	46.7% (558/1196)	
CSS				0.441
Alive	60.2% (539/896)	62.7% (188/300)	60.8% (727/1196)	
Dead due to cancer	39.8% (357/896)	37.3% (112/300)	39.2% (469/1196)	
Survival, months	38.8 ± 37.1	38.8 ± 37.1	38.9 ± 36.3	0.866

*Abbreviations*: *AJCC* American Joint Committee on Cancer, *TNM* tumor–node–metastasis, *OS* overall survival, *CSS* cancer-specific survival

### Univariate and multivariate analyses of prognostic factors in the training cohort

The predictors of OS and CSS identified from univariate and multivariate analysis of the training cohort are shown in Tables 2 and 3.

**Table 2:** Univariate and multivariate analyses of factors associated with OS in the training cohort

Variable	Univariate analysis		Multivariate analysis	
	**HR (95% CI)**	***P* value**	**HR (95% CI)**	***P* value**
Age, years				
0–55	Reference		Reference	
56–67	1.6 (1.2, 2.0)	0.001	1.2 (0.9, 1.6)	0.206
≥ 68	2.7 (2.1, 3.5)	0.001	1.7 (1.3, 2.2)	< 0.001
Sex				
Female	Reference		Reference	
Male	1.1 (0.9, 1.3)	0.336	1.3 (1.1, 1.6)	0.005
Tumor size, mm				
< 35	Reference		Reference	
≥ 35	4.3 (3.4, 5.4)	< 0.001	1.5 (1.2, 2.0)	0.002
Laterality				
Right	Reference		Reference	
Left	1.1 (0.8, 1.3)	0.663	1.2 (1.0, 1.6)	0.077
Grade				
G1	Reference		1	
G2	2.0 (1.3, 2.9)	< 0.001	1.5 (1.0, 2.2)	0.066
G3	8.8 (6.6, 11.7)	< 0.001	4.8 (3.3, 6.9)	< 0.001
G4	10.8 (7.8, 15.0)	< 0.001	5.2 (3.5, 7.8)	< 0.001
Pathology				
8013/3 large cell neuroendocrine carcinoma	Reference		Reference	
8041/3 small cell carcinoma	1.8 (1.2, 2.9)	0.006	1.5 (0.9, 2.4)	0.091
8240/3 carcinoid tumor	0.1 (0.1, 0.2)	< 0.001	0.7 (0.5, 1.1)	0.173
8244/3 mixed adeno-neuroendocrine carcinoma	0.8 (0.5, 1.4)	0.472	1.5 (0.9, 2.5)	0.158
8246/3 neuroendocrine carcinoma	0.7 (0.5, 0.9)	0.009	1.3 (1.0, 1.8)	0.094
Operation				
No operation	Reference		Reference	
Local tumor excision	0.0 (0.0, 0.1)	< 0.001	0.3 (0.1, 1.1)	0.066
Curative	0.5 (0.4, 0.8)	0.005	0.7 (0.5, 1.2)	0.181
Chemotherapy				
No	Reference		Reference	
Yes	3.2 (2.6, 3.8)	< 0.001	0.8 (0.6, 1.0)	0.029
AJCC TNM stage (8th)				
I	Reference			
IIA	2.5 (0.9, 7.3)	0.095		
IIB	2.2 (0.9, 4.9)	0.068		
IIIA	6.0 (2.2, 16.6)	< 0.001		
IIIB	4.4 (2.3, 8.4)	< 0.001		
IV	15.6 (8.3, 29.4)	< 0.001		
AJCC T stage (8th)				
T1	Reference		Reference	
T1a	0.1 (0.0, 0.2) < 0.001	< 0.001	1.7 (0.5, 5.5)	0.387
T1b	0.1 (0.0, 0.2) < 0.001	< 0.001	0.4 (0.1, 1.8)	0.248
T2	0.2 (0.1, 0.4) < 0.001	< 0.001	0.7 (0.3, 2.1)	0.575
T3	0.3 (0.1, 0.8)	0.012	0.9 (0.3, 2.3)	0.776
T4	0.5 (0.2, 1.1)	0.093	0.9 (0.3, 2.3)	0.827
AJCC N stage (8th)				
N0	Reference		Reference	
N1	2.5 (1.9, 3.3)	< 0.001	1.9 (1.4, 2.6)	< 0.001
AJCC M stage (8th)				
M0	Reference		Reference	
M1	4.5 (3.7, 5.5)	< 0.001	3.5 (2.8, 4.4)	< 0.001

*Abbreviations*: *AJCC* American Joint Committee on Cancer, *TNM* tumor–node–metastasis, *HR* hazard ratio, *CI* confidence interval

**Table 3:** Univariate and multivariate analyses of factors associated with CSS in the training cohort

Variable	Univariate analysis		Multivariate analysis	
	**HR (95% CI)**	***P* value**	**HR (95% CI)**	***P* value**
Age, years				
0–55	Reference		Reference	
56–67	1.6 (1.2, 2.1)	0.001	1.2 (0.9, 1.6)	0.26
≥ 68	2.3 (1.8, 3.0)	0.001	1.4 (1.0, 1.9)	0.026
Sex				
Female	Reference		Reference	
Male	1.1 (0.9, 1.3)	0.507	1.3 (1.0, 1.6)	0.021
Tumor size, mm				
< 35	Reference		Reference	
≥ 35	5.5 (4.2, 7.1)	< 0.001	1.6 (1.2, 2.1)	0.001
Laterality				
Right	Reference		Reference	
Left	1.1 (0.9, 1.4)	0.393	1.4 (1.1, 1.8)	0.017
Grade				
G1	Reference		Reference	
G2	3.2 (2.0, 5.1)	< 0.001	2.2 (1.3, 3.5)	0.002
G3	14.6 (10.0, 21.4)	< 0.001	7.0 (4.4, 11.0)	< 0.001
G4	17.8 (11.8, 26.9)	< 0.001	7.5 (4.6, 12.2)	< 0.001
Pathology				
8013/3 large cell neuroendocrine carcinoma	Reference		Reference	
8041/3 small cell carcinoma	1.8 (1.1, 2.8)	0.015	1.4 (0.8, 2.2)	0.201
8240/3 carcinoid tumor	0.1 (0.1, 0.2)	< 0.001	0.6 (0.4, 1.0)	0.072
8244/3 mixed adeno-neuroendocrine carcinoma	0.9 (0.5, 1.4)	0.569	1.4 (0.8, 2.5)	0.18
8246/3 neuroendocrine carcinoma	0.6 (0.5, 0.9)	0.005	1.2 (0.9, 1.7)	0.213
Operation				
No operation	Reference		Reference	
Local tumor excision	0.0 (0.0, 0.1)	< 0.001	0.6 (0.1, 7.2)	0.723
Curative	0.5 (0.3, 0.8)	0.004	0.8 (0.5, 1.2)	0.263
Chemotherapy				
No	Reference		Reference	
Yes	3.8 (3.1, 4.7)	< 0.001	0.8 (0.6, 1.0)	0.034
AJCC TNM stage (8th)				
I	Reference			
IIA	14.8 (1.5, 142.0)	0.02		
IIB	10.2 (1.2, 84.5)	0.032		
IIIA	39.9 (4.5, 357.1)	< 0.001		
IIIB	34.9 (4.9, 249.8)	< 0.001		
IV	143.2 (20.1, 1021.2)	< 0.001		
AJCC T stage (8th)				
T1	Reference		Reference	
T1a	0.0 (0.0, 0.1) < 0.001	< 0.001	0.4 (0.1, 2.6)	0.324
T1b	0.0 (0.0, 0.2) < 0.001	< 0.001	0.3 (0.0, 1.5)	0.139
T2	0.1 (0.1, 0.4) < 0.001	< 0.001	0.7 (0.3, 2.0)	0.528
T3	0.3 (0.1, 0.7)	0.007	0.8 (0.3, 2.0)	0.617
T4	0.5 (0.2, 1.2)	0.111	0.9 (0.3, 2.3)	0.796
AJCC N stage (8th)				
N0	Reference		Reference	
N1	3.4 (2.5, 4.7)	< 0.001	2.2 (1.5, 3.1)	< 0.001
AJCC M stage (8th)				
M0	Reference		Reference	
M1	5.8 (4.7, 7.3)	< 0.001	4.0 (3.1, 5.1)	< 0.001

*Abbreviations*: *AJCC* American Joint Committee on Cancer, *TNM* tumor–node–metastasis

**Fig. 1:**
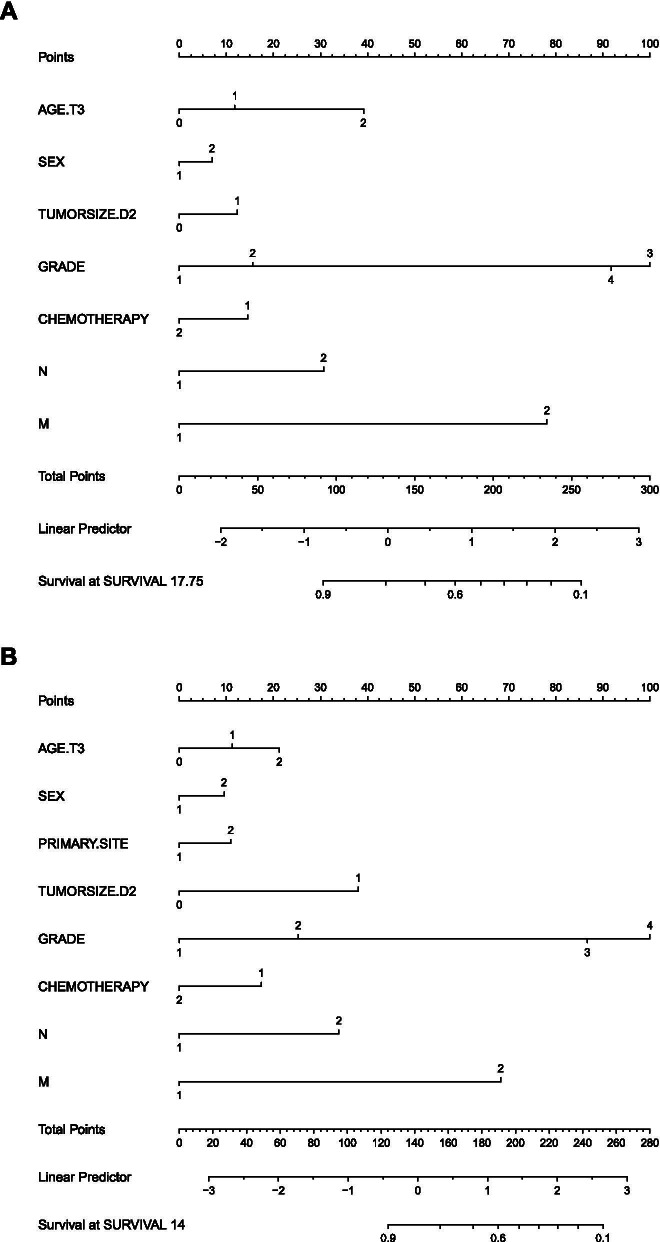
OS and CSS associated nomograms for colon NENs patients. **A** OS nomogram for colon NENs patients. **B** CSS nomogram for colon NENs patients

Univariate analysis indicated that age, sex, tumor size, grade, surgery, chemotherapy, AJCC TNM stage (8th), N stage, and M stage were significantly associated with the OS rate (Table 2). In multivariable survival analysis, age ≥ 68 years (*P* < 0.001), sex (*P* < 0.05), tumor size (*P* < 0.05), grade (*P* < 0.001), chemotherapy (*P* < 0.05), N stage (*P* < 0.001), and M stage (*P* < 0.001) were identified as independent prognostic factors associated with the OS rate. Moreover, age, sex, tumor size, laterality, grade, chemotherapy, AJCC TNM stage (8th), N stage, and M stage were identified as predictors of CSS by univariate analysis. However, only age ≥ 68 years (*P* < 0.05), sex (*P* < 0.05), laterality (*P* < 0.05), tumor size (*P* < 0.05), grade (*P* < 0.001), chemotherapy (*P* < 0.001), N stage (*P* < 0.001), and M stage (*P* < 0.001) were significant factors identified in multivariate analysis and were further used to develop a CSS nomogram (Table 3, Fig. 1B).

### Construction and validation of nomograms

OS and CSS nomograms were constructed based on independent prognostic factors identified by multivariable analysis (Fig. 1). By summing the scores for each selected variable, the probability of a patient’s survival can be easily obtained from the nomogram.

**Fig. 2:**
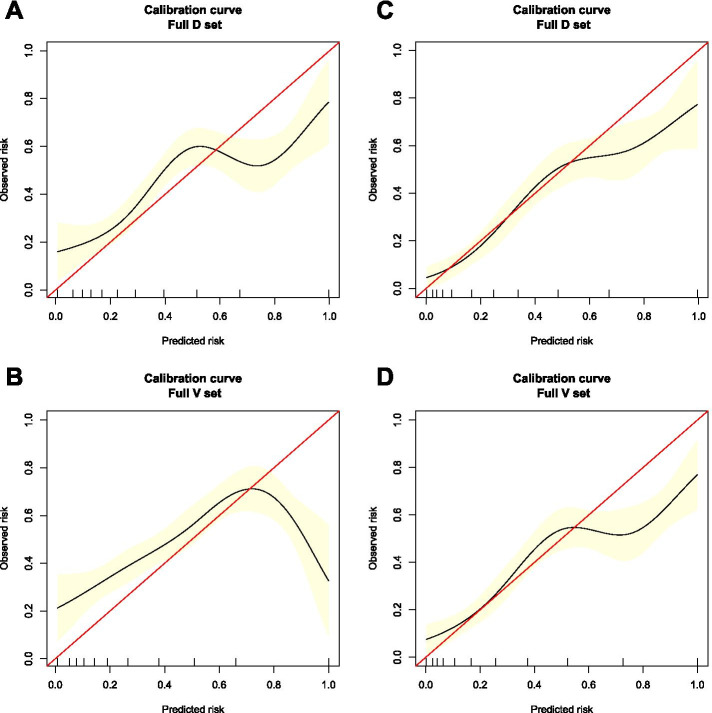
Calibration curves of the OS nomogram in the (**A**) training cohort and (**B**) validation cohort. Calibration curves of the CSS nomogram in the (**C**) training cohort and (**D**) validation cohort. The *x* axis represents the nomogram-predicted survival rate, whereas the *y* axis represents the actual survival rate

**Fig. 3:**
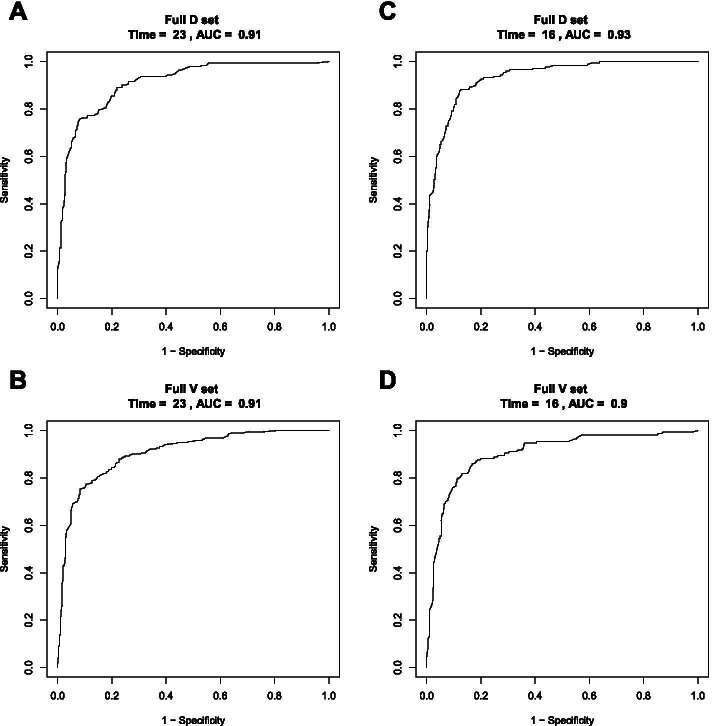
AUCs of the OS nomogram in the (**A**) training cohort and (**C**) validation cohort. AUCs of the CSS nomogram in the (**B**) training cohort and (**D**) validation cohort

We compared the predictive accuracy of our nomograms with that of the 8th AJCC TNM classification in the training cohort. The C-index of the OS nomogram was 0.8347 (95% confidence interval [CI], 0.8171–0.8523), which was higher than the C-index of the 8th AJCC TNM staging system (0.7159; 95% CI, 0.6762–0.7557). The C-index of our CSS nomogram was 0.8668 (95% CI, 0.0.8506–0.883), which was also superior to that of the 8th AJCC TNM staging system (0.7366; 95% CI, 0.6955–0.7776). These results demonstrated that our nomograms had superior survival predictive ability compared with the AJCC TNM staging system. To confirm the predictive power of the nomograms, further applications were made in the validation cohort, yielding a C-index of 0.8345 (95% CI, 0.8044–0.8646) and 0.8209 (95% CI, 0.7808–0.861) for the OS nomogram and CSS nomogram, respectively. The calibration curve revealed the agreement between the predicted and actual survival (Fig. 2). In addition, the area under the ROC curve (AUC) was high for both the training and validation cohorts (Fig. 3).

## Discussion

With widespread use of gastrointestinal endoscopy for cancer screening and increased public health awareness, the incidence of neuroendocrine tumors has been increasing in recent years [1]. The SEER study in the USA showed an age-adjusted incidence rate of gastroenteropancreatic neuroendocrine tumors of 3.56 per 100,000 inhabitants (2000–2012) [2]. It is well known that the primary site of NENs is an important prognostic factor for survival [2]. However, most previous studies have analyzed rectal NENs and colon NENs as if they originate from the same primary site [18, 19]. Nevertheless, it has become clear that colon NENs are a different disease from rectal NENs. Rectal NENs are commonly (but not exclusively) small and generally of low to intermediate grade (grades 1 [G1] or 2 [G2]), whereas colon NENs are often aggressive, poorly differentiated, and of higher grade (G3) [20]. Additionally, the OS rate of patients with colon NENs is significantly lower than that of patients with rectal NENs [21]. Therefore, it is necessary to perform separate statistical analysis for colon NENs.

Colon NENs are extremely rare, constituting only 1% of all colon neoplasms and < 11% of gastrointestinal NENs [22]. There are few studies related to colon NENs. According to Smith et al. [23], high-grade colorectal NECs are very aggressive tumors with poor prognosis. Patients have a slightly better prognosis if they do not have metastatic disease, if they have an adenocarcinoma component within the tumor, or if they respond to chemotherapy [23]. Unlike patients with non-colorectal non-neuroendocrine liver metastases [24–26], surgery, especially in the presence of metastatic disease, may not provide any survival benefit for most patients [23]. Fields et al. [27] demonstrated that the total number of positive lymph nodes was an independent predictor of survival in patients with colon NENs. Namely, the prognosis differed between patients with no positive lymph nodes, 1 positive lymph node, 2 to 9 positive lymph nodes, and 10 or more positive lymph nodes [27].

The most commonly used predictive system for colon NENs is the AJCC TNM classification, which includes three clinical parameters: tumor size (T), lymph node status (N), and distant metastasis (M). Studies have shown that for other types of solid tumors, multiple factors affect the prognosis of tumors [28, 29], but the TNM staging system has the most important prognostic value. However, for GEP-NENS, tumor differentiation is the most important prognostic indicator for disease course and progression [30].

In the present study, we developed and validated new nomogram models for predicting the OS and CSS in patients with colon NENs using the SEER public database, which includes the largest sample size of colon NENs to date. The nomogram incorporated independent prognostic factors associated with OS and CSS, which had been identified in the multivariable analysis, including age, sex, tumor size, grade, chemotherapy, N stage, and M stage.

According to our nomogram, an age ≥ 68 years and tumor size ≥ 35 mm were significantly associated with poor survival, and patients with lymph node metastasis and distant metastasis had a shorter survival time than those without metastasis. Furthermore, the classical T stage did not show independent prognostic significance in the nomogram model; instead, tumor grade showed a dramatic impact on prognosis. These results clarify the difference between prognosis predicted using the AJCC TNM staging system and prognosis based on tumor grade status of NENs.

In addition, this study included chemotherapy as a treatment strategy in the analysis. Thus, clinicians can use the total score provided by the nomograms constructed in this study to individualize treatment for patients with colon NENs and distinguish subgroups of patients at different levels of risk, thereby avoiding overtreatment in lower-risk patients and pursuing more aggressive treatment and close follow-up in higher-risk patients. In this study, chemotherapy was administered mainly in patients with high grade or late TNM staging (Supplement 1), with the potential to improve OS and CSS rates. Chemotherapy is recommended in patients with high tumor grade and advanced disease, who tolerate the side effects of chemotherapy. The nomogram can predict the prognosis of patients with colon NENs more accurately and provide clinicians with more useful information for developing targeted treatments.

The present study still had some limitations. First, neuroendocrine biomarkers, such as chromogranin A (CgA), synaptophysin (Syn), and CD56, were not available in the SEER database. Therefore, it was impossible to evaluate these parameters and integrate them into the nomogram. Moreover, in the SEER database, Ki-67 index was classified as well differentiated, moderately differentiated, and poorly differentiated/undifferentiated, which is why it was used as a categorical variable in the nomogram; however, Ki-67 is a continuous variable in clinical practice. Therefore, the use of a continuous Ki-67 index variable may be more useful in developing nomograms and predicting outcomes more accurately. Second, the SEER database did not contain detailed data regarding chemotherapy regimens, which restricted us from further evaluating the impact of different drug treatments on the survival of patients with colon NENs. Third, for the validation of nomograms, both internal and external validation cohorts are recommended. Due to the rarity of colon NENs, the number of cases collected in our center in Jiangsu province from 2010 to 2019 was too small to perform external validation, and that is why only internal validation could be performed in this study. Moreover, it was difficult to achieve no difference in every indicator because of the large sample size. Over time, we plan to collect more patients and variables to further refine the nomogram. Despite these inherent limitations, our prognostic model still provides a helpful tool for clinicians to ensure better decision making and prognosis estimation.

## Conclusion

In conclusion, we identified seven independent prognostic factors of survival, including age, sex, tumor size, grade, chemotherapy, N stage, and M stage, and developed new nomograms to predict OS and CSS in patients with colon NENs using the SEER data. Our nomograms demonstrated a better survival predictive ability than the 8th AJCC TNM staging system, and thus they can be valuable tools for individualized clinical decisions.

## Supplementary Information

**Additional file 1. Supplement 1.** Characteristics of histological differentiation and TNM staging (8th edition) in colon NENs patients receiving chemotherapy.

## Data Availability

The datasets supporting the conclusions of this article are available in the SEER database at https://seer.cancer.gov/seerstat/.

## Abbreviations

NENs: Neuroendocrine neoplasms
NET: Neuroendocrine tumor
NEC: Neuroendocrine carcinoma
MiNEN: Mixed neuroendocrine non-neuroendocrine neoplasm
MANEC: Mixed adeno-neuroendocrine carcinoma
OS: Overall survival
CSS: Cancer-specific survival
AJCC: American Joint Committee on Cancer
TNM: Tumor–node–metastasis
SEER: Surveillance, Epidemiology, and End Results
WHO: World Health Organization
ICD-O-3: International Classification of Diseases for Oncology, Third Edition
CI: Confidence interval
C-index: Concordance index
ROC: Receiver operating characteristic
AUC: Area under the time-dependent ROC curve
